# Neighborhood crime, disorder and substance use in the Caribbean context: Jamaica National Drug Use Prevalence Survey 2016

**DOI:** 10.1371/journal.pone.0224516

**Published:** 2019-11-22

**Authors:** Erica Ann Felker-Kantor, Colette Cunningham-Myrie, Lisa-Gaye Greene, Parris Lyew-Ayee, Uki Atkinson, Wendel Abel, Pernell Clarke, Simon G. Anderson, Katherine P. Theall

**Affiliations:** 1 Department of Global Community Health and Behavioral Sciences, School of Public Health and Tropical Medicine, Tulane University, New Orleans, Louisiana, United States of America; 2 Department of Community Health and Psychiatry, University of the West Indies, Mona, Kingston, Jamaica; 3 Mona GeoInformatics Institute, University of the West Indies, Mona, Kingston, Jamaica; 4 National Council on Drug Abuse, Ministry of Health, Kingston, Jamaica; 5 Organization of American States, Inter-American Drug Abuse Control Commission, Washington, District of Columbia, United States of America; 6 The George Alleyne Chronic Disease Research Centre, Caribbean Institute of Health Research, University of the West Indies, Cave Hill, Barbados; George Washington University, UNITED STATES

## Abstract

The purpose of the study was to examine the role of objective and subjective measures of neighborhood crime and disorder on substance use among a nationally representative sample of 4525 Jamaicans aged 12–65 years. Log-Poisson models with generalized estimating equations were used to estimate relative risks (RR) and 95% confidence intervals (CI). A test of interaction was used to determine presence of effect modification by sex. Approximately 39% of the study population reported past-month alcohol use; 10% past-month tobacco use; and 15% past-month marijuana use. In fully adjusted models, past-month alcohol and tobacco use were associated with perceived neighborhood disorder (p<0.05). The likelihood of alcohol use was 1.12 (95%CI:1.04, 1.20) times greater among participants who perceived higher neighborhood disorder. The likelihood of tobacco use was 1.22 (95%CI: 1.01, 1.46) times greater among participants who perceived higher neighborhood disorder. A significant test for interaction in adjusted models (P<0.2) suggested that the associations between substance use and perceived neighborhood disorder varied by sex. Examination of stratified models indicated that the role of perceived neighborhood disorder on alcohol and tobacco consumption varied among females, but not males. Females who perceived higher levels of neighborhood disorder had an increased likelihood of past-month alcohol and tobacco use (RRa:1.25 95%CI:1,07, 1.45; RRa:1.73 95%CI: 1.10, 2.67). Objective neighborhood crime measures were not associated with alcohol, tobacco, or marijuana use. The study findings provide evidence for the importance of considering subjective and objective neighborhood measures when examining relations with health outcome and demonstrate that perceptions of context and contextual exposures are not uniform across populations within neighborhoods. Interventions focused on building community trust and social cohesion (e.g. neighborhood community watch groups) and greening of blighted or abandoned spaces may help increase the sense of safety and order, reducing stress and maladaptive coping such as substance use.

## Introduction

Dependence on alcohol and drugs can have deleterious effects on the psychological and physical well-being of individuals[1, 2]. Substance use has been associated with many adverse and long-term health outcomes including heart disease, liver failure, weakened immune system, lung disease, and mental confusion[3, 4]. Substance use also has psychosocial consequences such as breakdown of relationships and may lead to other risk behaviours (i.e. unprotected sex, etc.) by impairing decision-making and weakening the ability to process social cues[5, 6]. According to the 2016 global burden of disease report, alcohol use disorders are the most prevalent of all substance use disorders worldwide with an estimated age-standardised prevalence rate of 1320.8 per 100,000 population[7]. Marijuana and opioid dependence are the most common drug use disorders with an estimated 22.1 million cases and 26.8 million cases per year; and despite reductions in global smoking rates, tobacco use remains a leading risk factor for early death in more than 100 countries, accounting for 11.5% of global deaths in 2015 [7, 8].

A vast amount of research has documented risk and protective factors associated with substance use and misuse [1, 4, 9, 10]. While much of this research has focused on individual, peer, familial, and genetic correlates of substance misuse [11–14], a growing subset has focused on neighborhood environments as determinants of individual behaviors like substance use. Neighborhoods possess physical and social characteristics (e.g. crime, alcohol outlets, poverty, etc.,) that may impact individual health behaviors through various mechanisms, including physiological stress pathways [15]. Persistent exposure to noxious and threatening environments can induce a stress response which can impair health directly or indirectly through engagement in risk behaviors [16–18]. Substance use may be one method used to cope with social and environmental stressors such as neighborhood crime and violence [19].

Previous explorations of neighborhood-level determinants of substance use have primarily focused on indicators such as poverty, average household income, and economic disadvantage [20–24], as opposed to crime or safety specifically. Findings on the relation between these neighborhood exposures and substance use have been mixed, varying by type of substance and socioeconomic conditions [22, 23, 25–27]. A study by Galea et al., for example, reported that mean neighborhood income was associated with an increased likelihood of alcohol and marijuana use, but not cigarette use among a sample of adults in New York City [26]. Hill et al. reported a positive correlation between neighborhood disorder and heavy drinking [18], while Pollack and colleagues found higher rates of alcohol consumption in least deprived neighborhoods [28]. Inconsistent findings are due to many factors (i.e. study design, structural confounding, spatial misclassification, etc.,) including differences in how neighborhoods are operationalized.

While individual criminal behavior and substance use are highly correlated [29, 30], less is known about the relationship between neighborhood-level crime-related exposures and individual substance use [23, 31]. Crime and safety in one’s residential community, while highly correlated with poverty, may have a more proximal link to stress and health outcomes given their strong impact on physiologic systems. Exposure to violence has been found to influence the underlying neurobiological pathways related to threat perception, potentially altering an individual’s response to threat not only when exposed to violence, but also on a day-to-day basis [32]. In the case of neighborhood violence exposure, the perpetrator is not necessarily known nor the same person, triggering an adaptive constant vigilance response leading to a greater biologic toll.

Neighborhood crime and violence have been linked to several health outcomes across the lifespan, including obesity, hypertension, and adverse birth outcomes [33–37]. Studies have also positively linked subjective measures of neighborhood crime and lack of safety to smoking behaviors [38–43]. Research on the association of neighborhood crime-related exposures and alcohol and other drug use, however, is more limited [23, 26, 44, 45]. Also limited are studies that assess objective measures of neighborhood crime [41, 44, 46].

Many studies on neighborhood effects and health conceptualize place based on administrative boundaries (e.g. census tracts, zip codes) and aggregate measures of neighborhood characteristics (i.e. number of households below the poverty line, number of fast food outlets) (for a review, see Ross & Mirowsky, 2001) [16]. While defining neighborhoods in this way has advantages (i.e. standardized boundaries, quantification of area effects across a standard physical area, etc.,), this approach does not account for subjective perceptions of place, which may provide a different characterization of neighborhood social and physical environments than objective measures. Some researchers suggest that perceptions of one’s neighborhood may be even more important than objective, measurable factors in predicting health outcomes [47, 48]. Subjective interpretation of stressors plays an important role in determining the individual stress response [49]) and the ways in which individuals appraise and interpret neighborhood conditions. Results from studies using both objective and subjective measures of neighborhood characteristics have been mixed with varying levels of associations with substance use or other behavioral outcomes [46, 50–52]. The discordance among perceived and objective measures suggests that while they may be related, they are distinct concepts, which highlights the importance of using multiple methods to obtain a comprehensive understanding of crime and perceived safety.

In addition to perceived safety, a related measure—perceived neighborhood disorder—has also been linked to substance use behavior [21, 25, 53–56]. Neighborhoods with high levels of disorder have visible cues (i.e. graffiti, abandoned buildings, vandalism, crime, deviance, etc.,), which are often indicative of a breakdown in social control and weak community cohesion [57] and may evoke a physiological stress response [25] and therefore potential maladaptive coping behaviors [19].

The role of neighborhood crime and disorder on substance use may also differ by sex and gender. Research on crime and gender has shown that women have a greater fear of crime and perceive a greater risk of crime and victimization compared to men [58, 59]. Thus, it is likely that women and men perceive and experience neighborhood characteristics indicative of crime and safety differently which can influence their physiological stress responses and coping strategies. Results from studies that have investigated the role of gender as a potential effect modifier between neighborhood exposures and health outcomes have been mixed [26, 28, 60].While some evidence suggests that the health effect of residential context is larger for men, when specific environmental-level factors are examined residential context is related to health for both men and women. Further investigation of the differential health effects that contextual factors have on men and women is important for developing effective interventions.

The current study utilized data from the Jamaican National Drug Use Prevalence Survey to examine the role of both objective and subjective measures of neighborhood crime and disorder on alcohol, tobacco, and marijuana use among a nationally representative sample of Jamaican men and women aged 12–65 years. Compared to the United States, rates of drug and alcohol abuse in the Caribbean are relatively low [61], but recent epidemiological trends show a steady increase, especially in countries where the perceived risk is low and illicit substances are easily obtained [62]. However, data on substance use disorders in the Caribbean region is limited [3, 7, 62]. In 2016, as an effort to strengthen information on substance use among Caribbean populations, the National Council on Drug Abuse (NCDA) and the Organization of American States (OAS) in partnership with country governments launched an initiative to conduct drug use prevalence surveys in the region, the first survey (on which this study was based) was conducted in Jamaica [61].

The study adds to the scientific evidence base in several ways. First, the majority of studies investigating neighborhood effects have been conducted in the U.S., Canada, Europe. [23, 27, 63] The way in which neighborhoods are perceived and influence substance use behaviors in Jamaica likely differ from that of more developed western countries, especially considering recent changes (2015) to the country’s national drug act which loosened restrictions on personal use of marijuana [61]. Furthermore, crime remains a main public safety issue for Jamaicans and a significant threat to the country’s human and economic development [64]. Jamaica has homicide rates that are notably higher than both the regional and global averages falling within the top 10% globally with increases since 2015 [65, 66]. Second, the incorporation of perceived and objective markers of crime and disorder and their role on substance use behavior in a high crime context is also rare. Finally, contrary to many neighborhood studies that simply adjust for sex differences, we examine sex as a potential effect modifier.

## Materials and methods

### Study design and sample

In 2016, the NCDA in partnership with the OAS through the Inter-American Drug Abuse Control Commission (CICAD) conducted a cross-sectional National Drug Use Prevalence Survey in Jamaica to determine the prevalence and differential patterns of drug and alcohol use among Jamaicans aged 12–65 years. The survey explored issues such as access and exposure to drugs and alcohol, risk perception, and attitudes towards recent changes in marijuana legislation.

The sampling design was developed by experts from the Statistical Institute of Jamaica (STATIN) to yield prevalence substance use estimates at the national and parish levels. Jamaica’s administrative units consist of 3 counties (equivalent to U.S. states), 14 parishes (equivalent to U.S. counties), and 5,771 Enumeration Districts (EDs) (equivalent to a U.S. census tract). EDs are the smallest geographic unit into which Jamaica is divided to facilitate the collection of census and survey data. In urban areas the average ED size is approximately 150 dwellings and 100 dwellings in rural areas where dwellings are more dispersed.

The survey employed a stratified multi-stage cluster sampling design with EDs as the primary sampling units (PSU). The distribution of the EDs in the sample was proportional to the urban-rural distribution of EDs within each parish. Within the selected EDs, a random starting point was selected to identify the first household. The sampling interval was determined by the number of households in the ED and sampling continued until the ED was exhausted or 16 households had completed the survey. One individual aged 12–65 years was randomly selected in each household using the Kish methodology to participate in the survey. The final sample size was 4,623 individuals aged 12 to 65 years. Sampling weights were calculated by the probability of selection and non-response weights. Post-stratification weights based on parish level distributions of age and sex categories were applied to ensure that the distribution of the weighted sample matched the population distribution of the age and sex groups. A descriptive analysis of the survey results was conducted in 2016 [61].

In the present analysis, data from the Jamaica National Drug Use Prevalence Survey were geocoded using ArcGIS software (ESRI Inc, Redlands, CA) and merged with neighborhood-level data (enumeration district) obtained from Jamaica’s Mona Geoinformatics Institute’s (MonaGIS). Over 80% of participant addresses were matched to the year 2011 Enumeration Districts (ED) from the Statistical Institute of Jamaica (STATIN) with a minimum match score of 80%. Ninety-eight households were unable to be geocoded due to incomplete addresses, resulting in a final analytic sample of 4525 individuals nested in 758 ED’s.

The Jamaica National Drug Use Prevalence Survey was approved by the Ministry of National Security, Jamaica and all subjects provided informed consent. For participants aged 12–17 years, informed consent was obtained from the parent or guardian of the minor while assent was obtained from the minor. The current study, a secondary analysis, was approved by the University Hospital of the West Indies/University of the West Indies Ethics Committee.

### Data collection

Data were collected through face-to-face interviews administered by trained interviewers. A pre-coded questionnaire was uploaded to electronic tablets via the Survey-to-Go platform. This data collection method eliminated the need for traditional data entry and minimized interviewer data entry errors. The entered data was reviewed by supervisors and invalid entries were corrected. When it was not logistically feasible to conduct interviews with tablets, paper-based data collection was performed and transferred to the tablets.

### Measures

The outcome variables assessed were self-report binary measures on current alcohol, tobacco, and marijuana use with current use defined as use during 30 days before survey participation. Given the low self-reported prevalence of other drug use, we did not examine these at this time.

The main exposure variables included objective measures of both neighborhood violent and non-violent crime and a subjective measure of perceived neighborhood disorder. Neighborhood was defined using Enumeration Districts (EDs) which are Jamaican geographic administrative units equivalent to the U.S. census tract. Geocoded crime event locations from 2015–2016 were obtained from MonaGIS proprietary JAMNAV database. Crimes included murder, robbery, shootings, larceny, aggravated assault, and break-ins. Crimes were grouped according to level of violence. Violent crimes included murder, shootings, and aggravated assault. Non-violent crimes included larceny, robbery, and break-ins. Kernel density estimation (KDE) in ArcGIS was used to calculate the objective neighborhood violent and non-violent crime measures. KDE is a spatial method that averages the location of each data point with respect to neighboring data points. The process works by overlaying a grid of equally sized cells on the study area and calculating a density estimate based on the center points of each cell. Each distance between an incident and the center of a grid cell is weighted based on the kernel function and a search radius [67]. Parameter settings to estimate the density measures were based on crime mapping literature and included a search radius of .25 and .5 at 1 km intervals. KDE is useful for identifying patterns of crime and areas with higher and lower densities [68]. Both measures were dichotomized at the 75^th^ percentile as they were heavily skewed and presented non-normal distributions.

The subjective neighborhood measure, perceived neighborhood disorder, was adapted from Ross and Mirowskys’ neighborhood disorder scale [69]. The measure was constructed from participant responses to the following questions: “As far as you know, how much of the activities listed below are in your neighborhood?”: ‘drug dealing/trafficking’, ‘breaking and entering homes’, ‘scribbling graffiti on the walls, damaging cables or things of that kind’, ‘taking drugs in public places such as the street or square or on the block’, ‘armed robbery or mugging in the street’, ‘young people standing around or loitering at street corners or on the block’, ‘shootout and violence with firearms’. Response options were on a 4-point Likert scale ranging from ‘1 a great deal’ to ‘4 none’. Factor analysis was conducted to test validity. All items loaded on two factors (>0.4) and explained 63% of the total variance. The overall measure demonstrated good internal reliability (Cronbach’s alpha = 0.735). Responses were summed to create an index of perceived neighborhood disorder with scores ranging from 1–28. Given the skewed nature of the index, scores were categorized at the median into high and low perceived neighborhood disorder.

Socio-demographic characteristics including age (in years), sex (male/female), education (less than or equal to some secondary/greater than secondary), urban/rural residence, religion (Christian/Rastafarian/Other) household income (less than or equal to 50,000 Jamaican dollar/50,001–180,000/greater than 180,000), relationship status (single/married/divorced or separated) and employment (employed/unemployed/other) were assessed as potential confounders due to known associations with substance use outcomes.

### Statistical analysis

Univariate analysis (frequency distributions, means, and standard deviations) was performed to characterize the study sample and primary exposure and outcome variables. Pearson’s correlation coefficient was used to determine correlation between the objective crime density measures and subjective neighborhood disorder measure. Exploratory spatial mapping was also conducted to examine spatial patterns of substance use and crime events. Patterns of missingness in the data set were examined for any variables with >10%, to determine whether missingness revealed any pattern by confounding factors but no difference were detected. The majority of variables had <10% missing and were considered missing completely at random (MCAR).

SAS version 9.4 was used to fit a series of log-Poisson [70] models with generalized estimating equations, clustering by geographic area, and a compound symmetry working correlation structure in order to estimate the relative risk and 95% confidence intervals (CI) of current alcohol, marijuana, and tobacco use associated with living in a neighborhood characterized by high crime density and high perceived disorder controlling for sex, age, education, urbanicity, marital status, religion, and household income. Subsequently, we fit the same series of models including a test of interaction between neighborhood crime and perceived disorder and sex. Models with significant interaction terms (P<0.2) [69] were stratified to examine within-group differences. Model fit was assessed using QIC, the equivalent of Akaike’s Information Criterion (AIC) in generalized estimating equations. Alpha was set at p <0.05.

## Results

Table 1 presents weighted and non-weighted descriptive statistics for men, women, and the total study population. The study population was 45.2% male and average age was 35 years. More than two-thirds (67.6%) of participants were single and slightly more than half the sample (54.8%) were employed. More than half (56.1%) of participants had a monthly household income less than or equal to 50,000 Jamaican dollars (JAD). More than two-thirds (61.9%) of the sample had completed secondary education or higher. Most respondents identified as Christian (80.8%) and rural residence (58.5%) was slightly higher than urban. Alcohol was the most common substance consumed with slightly less than half the sample reporting current use (40.8%). Approximately 15% of the study sample reported current marijuana use and 10.8% were current smokers. Among neighborhood-level characteristics, mean non-violent crime density was 3.1 per km^2^ and mean violent crime density was 2.9 per km^2^ (not presented in Table 1).

**Table 1:** Un-weighted and weighted characteristics of study population in Jamaica’s National Drug Survey, 2016 (N = 4525).

	N = 4525	%/mean	%/mean weighted
**Sex**			
Male	2047	45.24	42.00
Female	2478	54.76	58.00
**Average Age (years)**	4525	35.12	34.29
**Marital status**			
Single	3054	67.64	65.51
Married	1357	30.06	32.79
Divorced/separated	104	2.3	1.69
**Residence**			
Urban	1879	41.52	48.36
Rural	2646	58.48	51.64
**Employment**			
Employed	2479	54.78	51.67
Unemployed	1411	31.18	31.63
Other	635	14.03	16.70
**Education**			
= <some secondary	1720	38.09	36.84
>some secondary	2796	61.91	63.16
**Religion**			
Christian	3657	80.82	80.04
Rastafarian	69	1.52	1.13
Other	799	17.66	18.82
**Ave. monthly income (hhold) JAD**			
< = 50,000	2330	56.16	49.88
50,001–180,00	1147	27.65	28.66
>181,000	353	8.61	11.40
**Current substance use (past 30 days)**			
Tobacco use	487	10.76	9.85
Alcohol use	1847	40.82	39.05
Marijuana use	696	15.38	13.11
**Neighborhood-level characteristics (N = 758)**			
**Perceived neighborhood disorder**	**N**	**%**	**% weighted**
Low	2009	44.40	40.82
High	2516	55.60	59.18
**Non-violent crime density per km**^**2**^			
High	1126	24.8	—
Low	3399	75.12	—
**Violent crime density per km**^**2**^			
High	1116	24.7	—
Low	3409	75.3	—
**Correlation of neighborhood measures**			
	**Perceived neighborhood disorder**		
	**Pearson’s**	**p-value**	
**Non-violent crime density**	0.17	<0.0001	—
**Violent crime density**	0.19	<0.0001	—

hhold.–household.

Note: totals may not equal 100% due to missing cases.

Participants reported perceived neighborhood disorder as high (55.6%) and low (44.4%).

**Fig 1:**
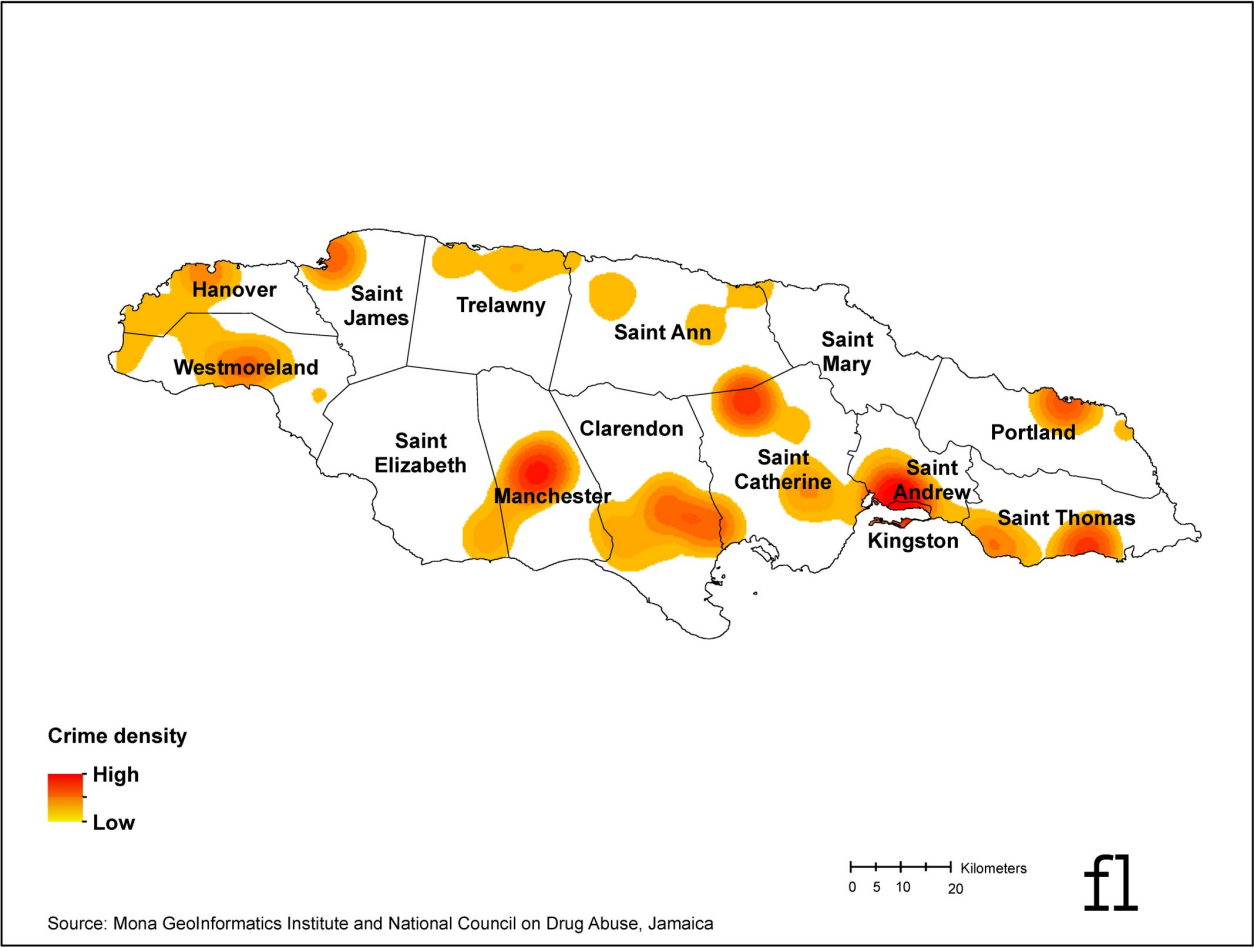
Crime density map, Jamaica 2015–2016. Geographic data source: Mona GIS, Jamaica. Published under a CC BY license, with permission from Mona GeoInformatics Institute, original copyright 2019.

**Fig 2:**
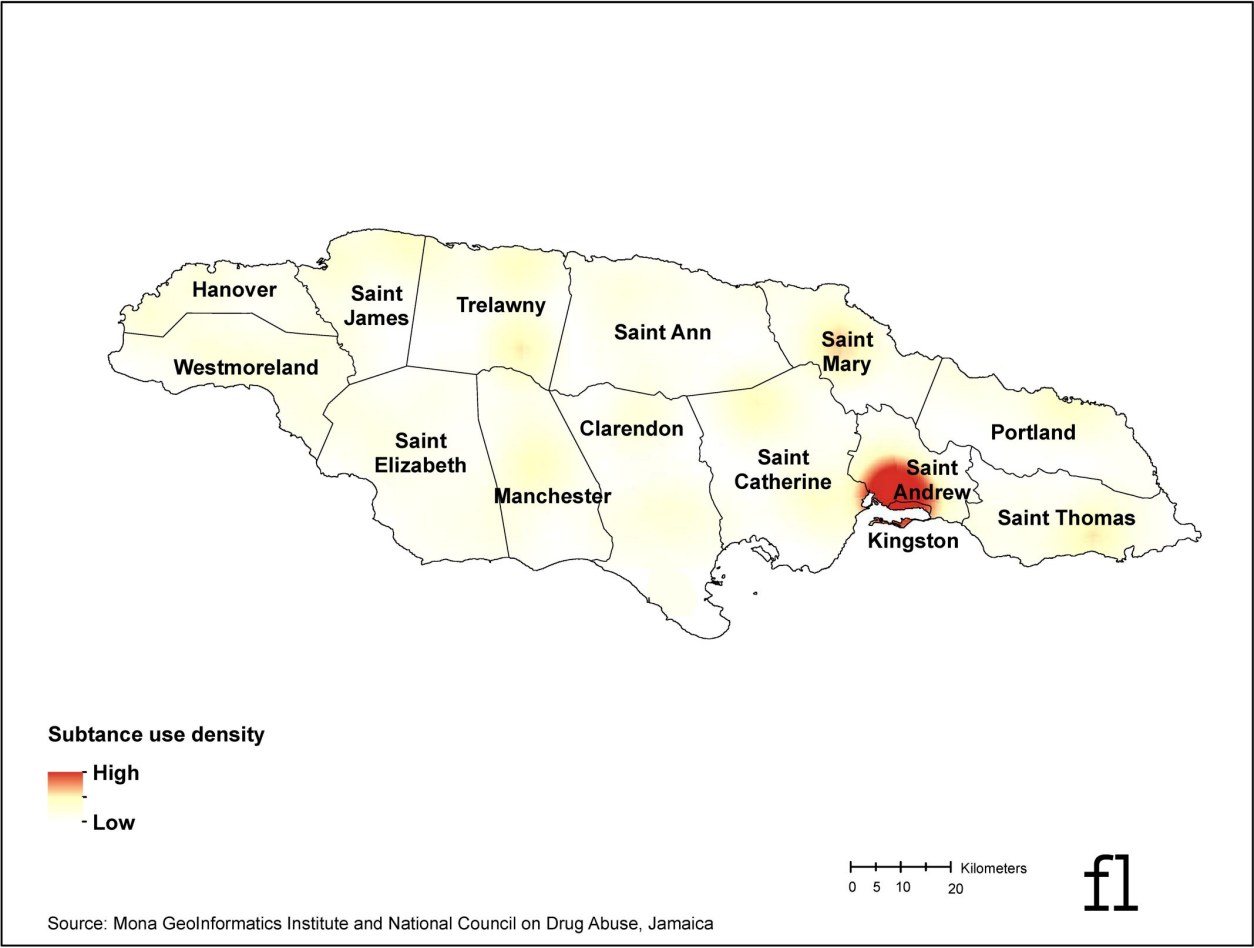
Current substance use density map, Jamaica 2016. Geographic data source: Mona GIS, Jamaica. Published under a CC BY license, with permission from Mona GeoInformatics Institute, original copyright 2019.

The Pearson correlation coefficient between perceived neighborhood disorder and non-violent crime density was 0.17 and 0.19 for violent crime density. Both were statistically significant (p<0.0001). Fig 1 is a kernel density map of all objective crime (non-violent and violent) events. Fig 2 is a kernel density map of current substance use hot spots (marijuana, alcohol, or tobacco).

Table 2 presents results from adjusted multivariable generalized *probit* models. Adjusting for demographic characteristics (sex, age, urbanicity, household income, marital status, education, and employment), perceived neighborhood disorder was statistically associated with current substance and tobacco use, but not marijuana use. Participants reporting high levels of neighborhood disorder were 1.12 (95%CI: 1.04,1.20) and 1.22 (95%CI: 1.01, 1.46) times more likely to report alcohol use and tobacco use in the past 30 days compared to those who perceived low neighborhood disorder. Neither objective neighborhood crime measure was associated with current marijuana, alcohol, or tobacco use in adjusted models.

**Table 2:** Adjusted relative risks and 95% confidence intervals between neighborhood crime densities and perceived neighborhood disorder and current substance use^1^.

	Marijuana Use		Alcohol Use		Tobacco Use	
	RR	95% CI	RR	95% CI	RR	95% CI
**Non-violent crime density per km**^**2**^						
Low	Ref.		Ref.		Ref.	
High	1.22	(0.99, 1.51)	1.02	(0.91,1.13)	1.03	(0.79,1.35)
**Violent crime density per km**^**2**^						
Low	Ref.		Ref.		Ref.	
High	1.16	(0.94,1.42)	1.02	(0.93,1.13)	1.01	(0.78,1.31)
**Perceived neighborhood disorder**						
Low	Ref.		Ref.		Ref.	
High	1.08	(0.92,1.28)	1.12	(1.04,1.20) **	1.22	(1.01,1.46) *

Ref.–referent category

^1^All models adjusted for age, sex, education, religion, employment, urbanicity, marital status and household income.

*P<0.05;

**P<0.01

A significant test for interaction in the adjusted models (P<0.2) suggested that the associations between substance use and perceived neighborhood disorder varied by sex. To further examine differences, we fit adjusted models stratified by sex. Table 3 presents adjusted relative risks and 95% CI’s for statistically significant associations in sex stratified adjusted models. The role of perceived neighborhood disorder on alcohol and tobacco consumption varied among females, but not males. Females who perceived higher levels of neighborhood disorder were 1.25 (95%CI: 1.07, 1.45) times more likely to be current alcohol users compared to females who perceived low neighborhood disorder. Similarly, females in high disordered neighborhoods were 1.73 times (95%CI: 1.10, 2.67) more likely to smoke compared to those living in low disordered areas. Marijuana use was not associated with perceived neighborhood disorder in stratified models.

**Table 3:** Adjusted relative risks and 95% confidence intervals between perceived neighborhood disorder and current alcohol and smoking use stratified by sex^1^.

	Alcohol Use				Tobacco Use			
	Male		Female		Male		Female	
**Perceived neighborhood disorder**	RR	95% CI	RR	95% CI	RR	95% CI	RR	95%CI
Low	Ref.								
High	1.05	(0.97,1.14)	1.25	(1.07,1.45) **	1.13	(0.93,1.37)	1.73	(1.10,2.67) **

Ref.–referent category

^1^All models adjusted for age, education, religion, employment, urbanicity, marital status and household income.

**P<0.01

## Discussion

While individual-level factors are important determinants of health behaviors and outcomes, accumulating evidence suggests that place is relevant for understanding variation in health outcomes because it comprises social relations and physical resources [15, 31]. A growing body of research on contextual effects and health has emerged from the United States and Europe; yet, there have been few place-based studies in developing countries. This study is one of the first to examine the impact of neighborhood conditions on substance use outcomes in the Caribbean region.

This study examined the potential role that neighborhood crime density and perceived neighborhood disoder had on susbtance use behaviors among a nationally representative sample of Jamaicains aged 12–65 years. We found that participants who perceived their neighborhood as more disordered were more likely to engage in alcohol use and tobacco use in the past 30 days compared to those who did not perceive their neighborhood as disordered. No association was detected between objective measures of crime and substance use [50]. Marijuana use was unrelated to both objective crime measures and perceived neighborhood disorder.

Although perceived neighborhood disorder and the objective crime density measures were significantly correlated, only perceived neighborhood disorder was related to substance use. This suggests that while the measures are related, they assess different constructs and is consistent with research showing that participants rate neighborhood environments differently from objective measures. Our findings further corroborate previous studies that have documented discordant results between subjective and objective neighborhood measures and health outcomes. This emphasizes the importance of personal observations of daily environments and supports the rational for including both subjective and objective measures in study designs [50, 51, 71].

The detected sex differences suggest that women and men perceive their environment differently with women experiencing a greater dose-response relationship between local environmental stressors and substance use. This aligns with other research which demonstrates that women have an increased susceptibility to neighborhood effects [72, 73]. One could argue, however, that women spend more time at home due to household and family responsibilities, making them more aware and exposed to the daily stressors of their local neighborhood social environment. Heightened stress exposure has been associated with numerous health outcomes including increased susceptibility to mental health problems and substance use. Women’s increased vulnerability to sexual assault may influence their perception of neighborhood safety and those who experience greater fear of assault or victimization may engage in substance use to manage their heightened stress. Future research incorporating time spent at home as well as mental health and past experiences with victimization in the neighborhood are needed to get a better understanding of the detected sex differences.

This study is not without limitations. These include the cross-sectional study design, and the self-report of substance use and perceived neighborhood disorder can be implicated in recall bias and same-source bias. To minimize self-report bias, validated and standardized instruments were used. Due to the cross-sectional design, we are unable to differentiate whether individuals who use substances choose to be in more disorganized areas or whether being in more disorganized neighborhoods influences substance use. To untangle this issue of selection vs influence, future studies should employ longitudinal designs. Another limitation is the potential for exposure misclassification in the objective crime measures due to the modifiable areal unit problem (MAUP) [66]. MAUP arises from aggregating point-based measures of spatial phenomena to arbitrarily defined geographic areas and the use of administrative boundaries as a proxy for neighborhood. Although EDs are the smallest unit of aggregation in Jamaica, they provide only a rough measure of neighborhood context. As a result, this could lead to incorrect specification of neighborhood-level exposures. Additionally, considering that criminal offenses are not always reported to the authorities an underrepresentation of actual crime events is plausible, which in turn, could lead to measurement error of the objective crime measures. Another limitation is the propensity for individuals to engage in substance use behavior outside of their neighborhood boundaries. This is especially plausible for youth who may want to hide substance use addictions from immediate family members. Finally, crime is inherently linked to poverty and unfortunately we did not have a measure of neighborhood poverty at the ED level; however, we did control for individual income.

Despite these limitations, our study makes several contributions to the literature on neighborhood effects and substance use. The examination of both objective crime and perceived neighborhood disorder in relation to substance use in a nationally representative sample of a developing country is a strength of this study. Contrary to many substance use studies, this study took a comprehensive view of substance use, examining multiple types of substance use. This allowed us to document differential relationships between different types of substance and different neighborhood characteristics. Additionally, our results provide further evidence for the importance of considering subjective and objective neighborhood measures when examining relations with health outcomes. Furthermore, the differential sex findings demonstrate that perceptions of context and contextual exposures are not uniform across populations within neighborhoods. The findings generated by this study may inform Jamaican policymakers to develop strategies that focus on increasing feelings of safety through community watch groups, community development such as greening and blight abatement, and creating social safety nets among neighbors. For maximum effect, it is critical that interventions are comprehensive addressing the concerns of women as well as men. Finally, future studies should look beyond the multilevel outcome-exposure associations and analyze the causal pathways through which contextual effects act on health behaviors (i.e., stress). A better understanding of how contextual factors trigger substance use behavior is critical for developing effective substance use interventions and policies.
